# Measuring axial length of the eye from magnetic resonance brain imaging

**DOI:** 10.1186/s12886-022-02289-y

**Published:** 2022-02-05

**Authors:** Stewart J. Wiseman, Andrew J. Tatham, Rozanna Meijboom, Graciela Muniz Terrera, Charlene Hamid, Fergus N. Doubal, Joanna M. Wardlaw, Craig Ritchie, Baljean Dhillon, Tom MacGillivray

**Affiliations:** 1 Centre for Clinical Brain Sciences, University of Edinburgh, 49 Little France Crescent, Edinburgh, EH16 4SB UK; 2 UK Dementia Research Institute, University of Edinburgh, Edinburgh, UK; 3 Edinburgh Imaging Facilities, University of Edinburgh, Edinburgh, UK; 4 NHS Lothian Princess Alexandra Eye Pavilion, NHS Lothian, Edinburgh, UK; 5 Centre for Dementia Prevention, University of Edinburgh, Edinburgh, UK

**Keywords:** Axial length, Biometry, MRI, Neurological

## Abstract

**Background:**

Metrics derived from the human eye are increasingly used as biomarkers and endpoints in studies of cardiovascular, cerebrovascular and neurological disease. In this context, it is important to account for potential confounding that can arise from differences in ocular dimensions between individuals, for example, differences in globe size.

**Methods:**

We measured axial length, a geometric parameter describing eye size from T_2_-weighted brain MRI scans using three different image analysis software packages (*Mango*, *ITK* and *Carestream*) and compared results to biometry measurements from a specialized ophthalmic instrument (*IOLMaster 500*) as the reference standard.

**Results:**

Ninety-three healthy research participants of mean age 51.0 ± SD 5.4 years were analyzed. The level of agreement between the MRI-derived measurements and the reference standard was described by mean differences as follows, Mango − 0.8 mm; ITK − 0.5 mm; and Carestream − 0.1 mm (upper/lower 95% limits of agreement across the three tools ranged from 0.9 mm to − 2.6 mm). Inter-rater reproducibility was between − 0.03 mm and 0.45 mm (ICC 0.65 to 0.93). Intra-rater repeatability was between 0.0 mm and − 0.2 mm (ICC 0.90 to 0.95).

**Conclusions:**

We demonstrate that axial measurements of the eye derived from brain MRI are within 3.5% of the reference standard globe length of 24.1 mm. However, the limits of agreement could be considered clinically significant. Axial length of the eye obtained from MRI is not a replacement for the precision of biometry, but in the absence of biometry it could provide sufficient accuracy to act as a proxy. We recommend measuring eye axial length from MRI in studies that do not have biometry but use retinal imaging to study neurodegenerative changes so as to control for differing eye size across individuals.

**Supplementary Information:**

The online version contains supplementary material available at 10.1186/s12886-022-02289-y.

## Key messages

**What was known**

1. Differences in eye geometry vary between individuals and it is important to correct for axial length to account for magnification error, however not all studies have access to precise optical biometry data.

**What this paper adds**

1. Measurement of axial length of the eye from brain MRI are repeatable and have good agreement with measurements obtained from optical biometry.

2. The eye provides a unique window to neural and vascular tissue, and ocular imaging are providing insights into the pathophysiology of dementia, stroke, multiple sclerosis and other diseases. Brain imaging studies that also acquire ocular imaging, but not biometry, can use this method to measure axial length.

## Background

Metrics derived from measurement of the human eye are increasingly used as biomarkers for the study of a wide range of vascular and neurological conditions including dementia, stroke and multiple sclerosis [1]. The eye provides a unique window to neural and vascular tissue, and new tools for ocular imaging are providing insights into the pathophysiology of these conditions by exploring associations with clinical outcomes and brain scanning features. Using devices such as optical coherence tomography (OCT) it is possible to image the optic nerve and retina and discern individual layers, such as the circumpapillary retinal nerve fiber layer (RNFL) and retinal ganglion cell layer, the sites of retinal ganglion cell axons and retinal ganglion cell bodies respectively. It is also possible to visualize the very smallest blood vessels including those that form the choroid [2].

When analyzing metrics derived from ocular imaging, it is important that the optical properties of the eye are considered. Differences in geometry such as globe size vary considerably between individuals and these differences are known to induce magnification effects and influence the absolute measurement of anatomical structures such as the RNFL. An important metric is axial length, which refers to the length of the eye from the anterior surface of the cornea to the internal limiting membrane (ILM) of the retina or retinal pigment epithelium (RPE), with the precise posterior measurement boundary dependent on device. Axial length is an important indicator of refractive state, with eyes with a short axial length (< 22 mm) typically hyperopic and eyes with long axial length (> 26 mm) typically myopic. Axial length has clinical applicability for eye disease, with myopia increasing the risk of diseases including open angle glaucoma [3–5] and retinal detachment [6], and hyperopia increasing the risk of diseases including angle closure glaucoma [7].

There may also be an association between axial length and structural measurements of the retina obtained using imaging devices such as OCT, which may confound studies examining OCT measurements as biomarkers of ocular and systemic disease [8] however results have been mixed and some studies have shown no such association [9]. Nevertheless, measurements derived from OCT can be affected by magnification effects related to axial length and the refractive properties of the eye [10]. In one study of 148 eyes, axial lengths shorter than 23.60 mm and longer than 25.55 mm required adjustment of RNFL thickness in order to account for ocular magnification [11].

Thicker RNFL, correcting for axial length, was associated with lower mean diffusivity as a marker of structurally intact white matter in brain regions involved in the neurodegenerative process of Alzheimer’s disease [12].

Axial length is typically assessed using specialized ophthalmic biometry instruments, such as the IOLMaster (Carl Zeiss Meditec AG, Jena, Germany) an optical biometer that relies on the principle of partial coherence interferometry [13]. In contrast, brain research mostly uses magnetic resonance imaging (MRI) as the primary imaging modality, and studies examining brain imaging and ocular parameters, for example using OCT, might not have access to instruments to perform precise ocular biometry. In the UK Biobank (https://www.ukbiobank.ac.uk/), 117,649 participants contributed eye data including 68,151 with retinal photography and OCT, but axial length was not measured using any modality.

The orbital contents are highly conspicuous on brain MRI, including T_2_-weighted scans where the vitreous humor is hyperintense to surrounding tissue, making axial length measurement a relatively straightforward task. In this study we measured eye axial length from T_2_-weighted brain MRI scans using three software packages and compared the results to measurements from a specialized instrument, considered the reference standard. We sought to assess agreement between MRI-derived data and the reference standard and ascertain whether the measures obtained from MRI can act as a proxy for axial length in studies where optical biometry is not performed.

## Materials and methods

### Study design

This was a retrospective, cross-sectional analysis of brain MRI data with available ocular biometry measurements.

### Participants

The subjects in this analysis were community-dwelling, cognitively-healthy individuals in mid-life (age 40 to 59 years) participating in the PREVENT Dementia Study [14], a study in 5 centres across the UK and Ireland investigating novel biomarkers for identifying early signs of degenerative brain disease (https://preventdementia.co.uk/). Study recruits had no known eye disease and were dementia-free at baseline. Fifty percent had a known family history of dementia at baseline and are therefore at increased risk of neuordegenerative disease and associated cognitive decline. The protocol for the PREVENT Dementia study has been described in detail previously [14]. Briefly, participants undergo brain and retinal imaging, blood tests and cognitive assessments. All participants gave written informed consent, and all procedures adhere to the tenets of the Declaration of Helsinki. The national study was reviewed by the London Research Ethics committee (12/LO/1023). Local imaging activities were reviewed by South East Scotland Research Ethics Committee (15/SS/0146) while the sponsors were the University of Edinburgh and NHS Lothian. All the participants in this analysis were from the Edinburgh site of the PREVENT Dementia cohort.

### Biometry

Axial length was measured with the IOLMaster 500 (Carl Zeiss Meditec AG, Jena, Germany), which utilizes a partial coherent interferometry technique with an infrared diode laser at the wavelength of 780 nm. Following the procedure recommended by the manufacturer, each subject was asked to fixate on the red internal light and the reflection of the alignment light was placed by the operator within the measurement circle on the instrument’s display screen. A signal-to-noise ratio (SNR) was given for each measurement and a value > 2.0 was considered as acceptable for measuring axial length. At least five readings of sufficient SNR were recorded to calculate a reliable mean value (within-subject standard deviation < 0.01 mm) that was used in subsequent analysis. Prior to each measurement session, the IOLMaster was verified as being operational and properly calibrated by measuring axial length of the Zeiss “test eye” and confirming that the result was within an accepted tolerance (±0.01 mm).

### Brain MRI

MRI used in this analysis were whole brain axial T_2_-weighted datasets (32 slices; 4 mm slice thickness; 1.2 mm slice gap; TR 1500; TE 80; flip angle 150; acquisition time 50 s) with in-plane voxel dimensions of 0.7 × 0.7 mm. All images were acquired on a Siemens 3 T research MRI scanner with consistent patient alignment in a 32-channel head coil and scan angulation aligned to the anterior-posterior commissure (AC-PC line) as per routine radiographic convention. Participants were instructed to keep their head as still as possible and immobilization pads were used in the head coil, but no specific instructions were given regarding eye rolling or keeping eyes open or closed as the brain was the main organ of interest, not the eyes. The scanner’s original Digital Imaging and Communications in Medicine image files were converted to Neuroimaging Informatics Technology Initiative format for subsequent analysis. All brain scans were reviewed by a radiologist and evaluated for quality prior to measurement by an experienced neuroimaging analyst.

### Eye axial length measurements

**Fig. 1:**
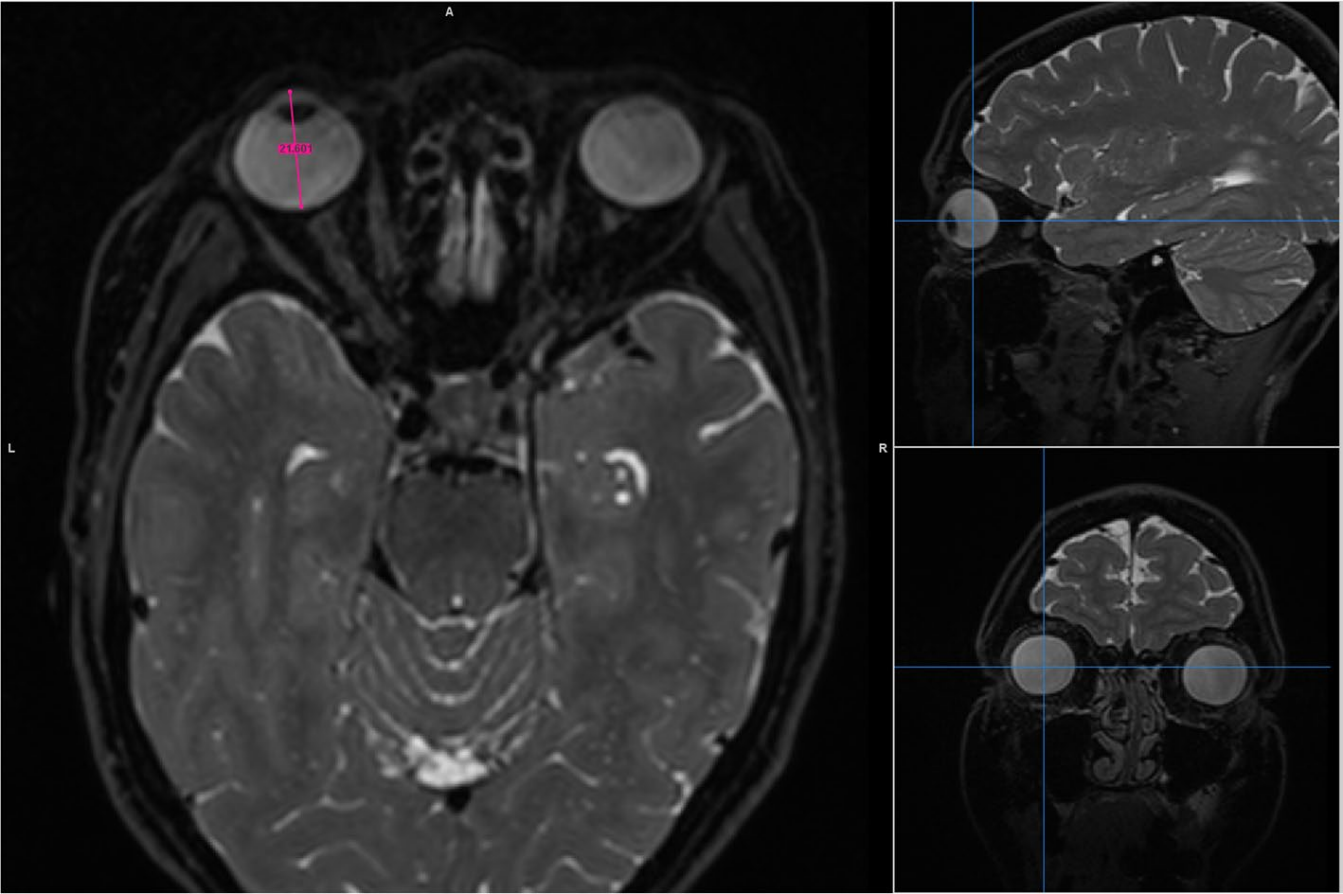
Procedure for finding the centre of the eye in three orthogonal planes on T2-weighted brain MRI. Example shown is the Mango image analysis tool – the main image component displays the brain in an axial orientation with two supplementary projections (sagittal top and coronal bottom) used to guide the user to the centre of the globe. Zoom tools were then used on the axial image to better visualize the anterior and posterior boundaries of the globe. Maximum eye axial length is then measured on the axial image (pink line). Supplementary Figs. 2, 3, 4, 5, 6, 7, 8 show examples using the Caresteam image analysis tool, magnified on the axial image to better show the anterior and posterior measurement boundaries

Maximum eye axial length was measured in both eyes (Fig. 1 and Supplementary Figs. 2, 3, 4, 5, 6, 7, 8). We used three different image analysis software tools, two of which are freely available for download: *Mango* (version 4.0.1; http://ric.uthscsa.edu/mango/) and *Insight Segmentation and Registration Toolkit* **(**ITK-SNAP, hereafter “ITK”) (version 3.6.0; http://www.itksnap.org). The third, *Carestream*, is a commercial radiology-grade picture archiving communication system (PACS) system (version 11.3.2.0020; https://www.carestream.com/).

Specific steps for conducting the measurements were as follows. First, for each of the three different image processing software tools, the image intensity per subject was set to the same value to ensure consistent visual conspicuity. All measurements were performed on identical workstations and under identical lighting conditions. Both eyes were measured but results from the right eye only are reported. All measurements were conducted in 2D mode. 3D reconstructions of the images available in this study may have caused erroneous measurements due to interpolation of the MRI datasets, which were not acquired with isotropic voxels. Next, the centre of the eye was localized in three orthogonal planes. When in the general central vicinity, the axial orientation was used to estimate the MRI slice more precisely with the longest axial dimension. Zoom functions were used to help identify tissue boundaries, and a measurement ruler placed such that it divided the globe in the axial view in two equal left and right halves. Anterior to posterior placement of the ruler (i.e. axial length) was as per Fig. 1, from the cornea anteriorly to the sclera at the posterior pole. Measurements per subject took less than one minute to complete.

### Statistical analysis

Two analysts conducted the measurements separately on the MRI data, repeating the procedure after a gap of one month, for a total of four measurements per subject (Supplementary Fig. 1). Each analyst’s measurements were averaged so that inter-rater reproducibility could be calculated. All statistical analyses were conducted in R Studio (version 1.1.442) (http://www.r-project.org/) [15]. Bland-Altman plots [16] were used to assess the level of agreement between the MRI measurements and the IOLMaster. Agreement between raters (reproducibility) of the MRI measures was assessed by the intraclass correlation coefficient (ICC) [17] (ICC estimates were calculated using the ‘irr’ package [18], based on a mean rating (*k* = 2), absolute agreement, 2-way random-effect model). Repeatability was assessed in the same way although the ICC estimates were based on a single rating, absolute agreement, 2-way mixed-effect model.

## Results

Data available at time of analysis included a total of 93 research participants (51 female) of mean age 51.0 ± SD 5.4 years (range 40 to 59 years). One participant had their IOLMaster axial length measurement conducted without removal of contact lenses. The biometry reference standard axial length in the right eye was 24.1 ± 1.2 mm (range 21.6 to 27.0 mm).

### Agreement: image analysis software packages to reference standard

**Fig. 2:**
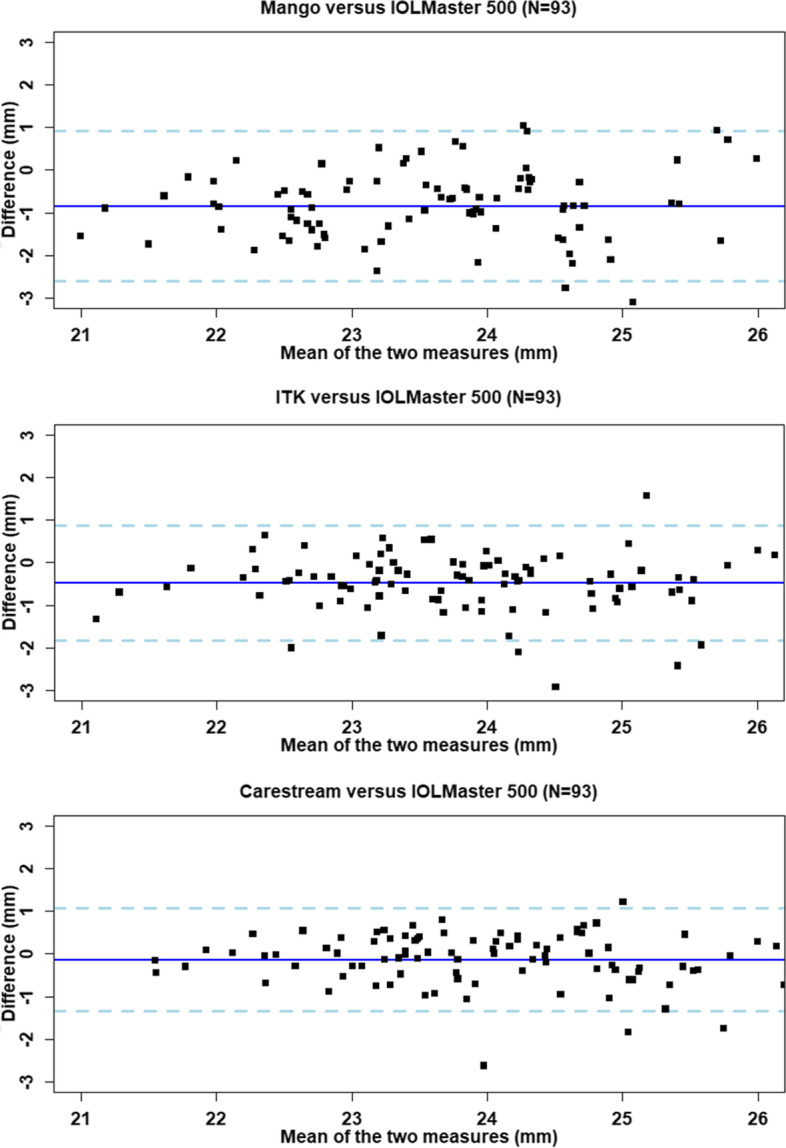
Bland-Altman plots showing agreement between right eye axial length measured in mm in Mango, ITK and Carestream versus the IOLMaster reference data (*N* = 93). Central solid lines indicate mean difference (MRI measures minus reference standard) and dashed lines indicated lower/upper limits of agreement (±1.96*SD). The points represent the individual deviation of each measurement compared with the reference

The level of agreement between the image analysis software and the IOLMaster are presented in Table 1 and Fig. 2. The mean differences are as follows, Mango: − 0.8 mm (95% confidence interval (CI) around the mean difference − 1.0 to − 0.6); ITK: − 0.5 mm (− 0.6 to − 0.3); and Carestream: − 0.1 mm (− 0.3 to − 0.0). The upper/lower 95% limits of agreement across the three tools ranged from 0.9 mm to − 2.6 mm. The Bland-Altman plots showed an even distribution of the differences indicating no systematic variance over the range of measurements, such that, even highly myopic eyes showed a similar relationship between MRI-derived axial length and biometry.

**Table 1:** Level of agreement between image analysis software tools and IOLMaster reference standard: mean differences (with 95% CI around the mean estimate) and lower/upper limits of agreement being ±1.96*SD (all data in mm; right eye)

Comparison to IOLMaster reference (mean 24.1 mm)	Average length	Lower limit of agreement	Mean difference			Upper limit of agreement
			95% CI lower	Mean	95% CI upper	
Mango	23.2	−2.6	−1.0	− 0.8	−0.6	0.9
ITK	23.6	−1.8	−0.6	− 0.5	− 0.3	0.9
Carestream	23.9	−1.3	−0.3	−0.1	− 0.0	1.0

### Reproducibility (inter-rater)

The level of agreement between the two analysts’ MRI measurements was described by mean differences as follows, Mango: 0.45 mm; ITK: − 0.03 mm; and Carestream: 0.18 mm (Table 2). ICC were between 0.65 (0.47 to 0.79) and 0.93 (0.86 to 0.95) depending on software tool.

**Table 2:** Inter-rater reproducibility of the MRI measures of axial lengths (mm) in the right eye: agreement by intraclass correlation coefficient

Software.	Analyst	Mean (SD)	Difference	ICC (95% CI)
Mango	1	23.3 ± 1.28		
	2	22.8 ± 1.43	0.45	0.65 (0.47 to 0.79)
ITK	1	23.6 ± 1.13		
	2	23.6 ± 1.19	−0.03	0.83 (0.75 to 0.88)
Carestream	1	24.0 ± 1.12		
	2	23.8 ± 1.11	0.18	0.93 (0.86 to 0.95)

### Repeatability (intra-rater)

The level of agreement between the first analyst’s one-month repeated MRI measurements is described by mean differences as follows, Mango: − 0.1 mm; ITK: 0.1 mm; and Carestream: − 0.2 mm (Table 3). ICC were between 0.93 (0.89 to 0.95) and 0.95 (0.86 to 0.97) depending on software tool. The level of agreement between the second analyst’s one-month repeated MRI measurements is described by mean differences as follows, Mango: 0.0 mm; ITK: − 0.2 mm; and Carestream: 0.1 mm (Table 3). ICC were between 0.90 (0.82 to 0.94) and 0.95 (0.93 to 0.97) depending on software tool.

**Table 3:** Intra-rater repeatability of the MRI measures of axial lengths (mm) in right eye measured by two analysts at two time points: agreement by intraclass correlation coefficient

Software	Analyst	1st time point	2nd time point	Difference	ICC (95% CI)
Mango	1	23.2 ± 1.29	23.3 ± 1.31	− 0.1	0.93 (0.89 to 0.95)
	2	22.8 ± 1.41	22.8 ± 1.51	0.0	0.90 (0.86 to 0.94)
ITK	1	23.6 ± 1.17	23.5 ± 1.13	0.1	0.95 (0.92 to 0.96)
	2	23.5 ± 1.22	23.7 ± 1.21	−0.2	0.90 (0.82 to 0.94)
Carestream	1	23.9 ± 1.13	24.1 ± 1.13	−0.2	0.95 (0.86 to 0.97)
	2	23.9 ± 1.13	23.8 ± 1.11	0.1	0.95 (0.93 to 0.97)

## Discussion

We demonstrate that measurement of axial length of the eye from brain MRI are repeatable and have good agreement with measurements obtained from optical biometry using the IOLMaster 500. This has utility in imaging studies that acquire brain and retinal image data but do not have access to a specialized instrument for measuring axial length. Mean differences in axial lengths derived from MRI relative to the reference standard were small and ranged from − 0.1 mm to − 0.8 mm (0.5 to 3.5% of the mean reference standard globe length of 24.1 mm). However, the upper/lower 95% limits of agreement (i.e., two standard deviations around the mean estimate) ranged from 0.9 mm to − 2.6 mm across the three image analysis tools, and a difference of almost 3 mm in a single patient could be considered clinically significant. Nonetheless, image magnification due to axial length variation is known to influence OCT [8] and OCTA [10] metrics and should be accounted for, and this could be particularly relevant in individuals at either end of the range of axial lengths as reported by Hirasawa et al. [11].

In brain image analysis it is routine practice to correct brain imaging biomarkers (such as white matter hyperintensities [19]) by total intracranial volume to account for differing head size between individuals. Similarly, correcting retinal imaging biomarkers such as circumpapillary RNFL thickness by axial length accounts for differing eye size, and use of routine image analysis tools to measure eye length from brain MRI scans provides a proxy. Here, we used one of the most commonly acquired brain MRI sequences, a whole brain T_2_-weighted scan which takes around 50 s to acquire on modern 3 T scanners, depending on acquisition parameters. Other sequences can be used for the purpose of measuring eye axial length, for instance Aiyekomogbon et al. [20] and Mendez-Gomez et al. [12] both used T_1_-weighted brain scans.

Specialized MRI sequences designed specifically to examine the orbital contents / optic nerve have better spatial resolution and are likely to deliver superior agreement to biometry than results presented here, but these specialized eye MRI sequences are not routinely acquired in most studies of brain disease.

As the resolution of MRI in this analysis was 0.7 mm, while the resolution of the IOLMaster is 0.01 mm, we do not suggest the measurement procedure presented here is a replacement for axial length obtained from optical biometry. Instead, we allow the reader to assess if the agreement is sufficient such that eye axial lengths from MRI can be used as a proxy in studies that do not include biometry.

Differences in axial length measurements between MRI and optical biometry may be due to different measurement boundaries. The IOLMaster calculates axial length by reflectance of infrared light from the RPE. Ultrasound may also be used but ultrasound uses the ILM, the inner most layer of the retina, as its posterior boundary, meaning measurements obtained from optical and ultrasound biometry are not interchangeable. Ultrasound also has the disadvantage of needing contact between a probe and the surface of the cornea, which requires topical anesthesia and can lead to distortion of the globe and inaccurate measurements if excessive pressure is applied. As infrared and ultrasound axial length measurements use different measurement boundaries, the IOLMaster makes an adjustment so its boundary is considered to be the ILM https://www.doctor-hill.com/iol-master/interpretation.htm. Neither the ILM nor RPE are visible on MRI, and therefore the posterior boundary used to determine axial length in the present study was the outer border of the sclera. A further potential reason for differences in measurements between techniques is possible differences in measurement axes. We employed a method whereby the maximum length of the globe was recorded, i.e. the anatomic or geometric axial length, whereas during optical biometry the patient fixates on a target meaning the distance measured is from the corneal to the fovea, i.e., the visual axis [21]. The optical axis is tilted an average of 5 degrees horizontally and 1 degree vertically relative to the anatomic axis, and is typically shorter than the anatomic axis.

A prior study [20] of 340 normal eyes from a Nigerian population that used T_1_-weighted brain MRI to measure axial length found right and left globes to be 23.32 ± 1.34 mm (range 22.0 to 24.7) and 23.29 ± 1.22 mm (range 22.10 to 24.51) respectively, results similar to ours, but did not assess agreement to a reference standard. In an approach similar to that presented here, Mendez-Gomez et al. [12] used two operators to measure axial length from brain scans in 104 elderly (mean age 80.8 years) people without dementia. They validated their MRI-derived axial length measurements in a subset of participants by correlation to IOLMaster biometry data (*r* = 0.89, *p* < 0.01) [12]. In a study of 3030 subjects aged between 20 and 89 years assessing globe position, axial length derived from T_1_-weighted MRI was 23.4 ± 0.8 mm for men and 22.8 ± 0.9 mm for women [22], results which when averaged across the sexes are to within 0.5 mm of our MRI measurements, and within the measurement error possible due to the inherent resolution of the scans. We did not analyse our data by sex differences due to the relatively small sample size.

MRI resolution is a potential limitation in our study as it was insufficient to visualize scleral thickness, which could be confounding as we cannot guarantee the same tissue boundary is selected when manually placing the software ruler tool on the MRI images. However, we sought to be consistent by placing the ruler on the visible outer scleral border in each case. Likewise, it is possible that a degree of measurement error could arise from inconsistencies in ruler placement on the cornea.

Repeatability of the IOLMaster itself is exceptional due to the use of laser reflectance technology, and far exceeds the repeatability achievable with MRI due to the comparatively lower spatial resolution of MRI. In a study of 26 healthy young subjects (mean age 19.3 years) repeatability of the IOLMaster was 0.0042 mm with 95% limits of agreement between 0.047 and − 0.039 mm [23].

MRI scans suffer from various artifacts including patient-related (involuntary/voluntary as well as pulsatile motion); processing artefacts like phase wrap; resolution artefacts; susceptibility artefacts and radiofrequency artefacts. Thus, images should be visually assessed before discerning measurements from them, particularly eye motion. We did not discard any scans due to artefacts.

Measurements obtained using Mango had the largest mean difference and widest confidence intervals indicating poorer performance when compared to ITK and Carestream. This could reflect issues with user interface as placing the measurement ruler was hampered slightly by the pointing device in Mango (a hand icon) which was subjectively considered by the analysts to be more difficult to place precisely compared to ITK and Carestream (both of which use an arrow pointing device).

Strengths of our study include use of three different image analysis software tools, repeated measurements to assess inter- and intra-rater agreement, use of Bland-Altman plots to assess agreement to the reference standard rather than correlation coefficients (correlation only reveals strength of relationship between two variables, not necessarily agreement) [16], and the use of ICC to assess reproducibility and repeatability because if the MRI method has poor repeatability the agreement to the IOLMaster is bound to be poor too [17]. We also conducted measures in the left eye; findings were very similar to the right eye presented here.

We used 2D scans in this study with 4 mm thick slices and a slice gap. It is possible that the maximum eye axial length fell within this slice gap in some subjects, but such a scenario is unlikely to have an impact on the measurements or our conclusions. Meanwhile, Nguyen et al. used ultra-high field strength 7 T MRI and a dedicated receiver coil placed close to the eye to assess several eye parameters including axial length, and repeated the axial length assessment in some participants with ultrasound biometry [24]. The use of 7 T MRI means improved signal-to-noise ratio and sub-millimeter spatial resolution. Whereas we under-estimated axial lengths with each of the three image analysis tools versus IOLMaster biometry, Nguyen *at el*. report larger axial lengths (on average by 0.5 mm) than those obtained by ultrasound biometry, concluding that the 95% confidence limits of agreement discrepancy (− 0.7 to 1.7 mm) was clinically significant.

To ensure consistency in measurements in the axial plane across a cohort, imaging should ideally be angled in the software tools to a recognized radiographic baseline such as the AC-PC line or rostrum-splenium line. Unfortunately, the use of 2D images in this study prohibited such consistent angulations. Angling 2D images necessitates conversion of acquired voxel dimensions (our scans were 5.2 × 0.7 × 0.7 mm) to approximate isotropic voxels using interpolation. This distorts anatomy and could result in erroneous measurements. However, as the actual scan acquisitions followed a standardized protocol with consistent radiographic planning, we are confident that potential differences due to subtly different head (and thus eyeball) angulations are minimal. We were unable to control for which subject had closed eyes during scanning as this data is not collected. For studies that acquire scans with isotropic voxels in 3D mode, we recommend consistent angulation in the other orthogonal planes prior to ruler placement in the axial plane.

Future efforts should include validating our results in other image analysis software packages, possible automation of the measurement procedure to accommodate large population studies like UK Biobank, and use of data from diseased populations with a larger spread of ages, including older ages where eye changes are common. We recommend measuring eye axial length from MRI in studies that do not have biometry but use OCT retinal imaging for example to study neurodegenerative changes to control for differing eye size across individuals, which will also minimize the burden of investigations for patients. Datasets that have MRI-derived axial length, specialized biometry and OCT should correct for the ocular magnification in OCT to test for a significant effect on the circumpapillary RNFL using each method.

## Supplementary Information

**Additional file 1.**

**Additional file 2.**

**Additional file 3.**

**Additional file 4.**

**Additional file 5.**

**Additional file 6.**

**Additional file 7.**

**Additional file 8.**

## Data Availability

The datasets generated and/or analysed during the current study are not publicly available due to limitations of ethical approval involving the patient data and anonymity but are available from the corresponding author on reasonable request.
