# Supplementary figures and images for: Measuring axial length of the eye from magnetic resonance brain imaging
Source: BMC Ophthalmol. 2022 Feb 5;22:54. doi: 10.1186/s12886-022-02289-y (PMC8817515; doi:10.1186/s12886-022-02289-y)

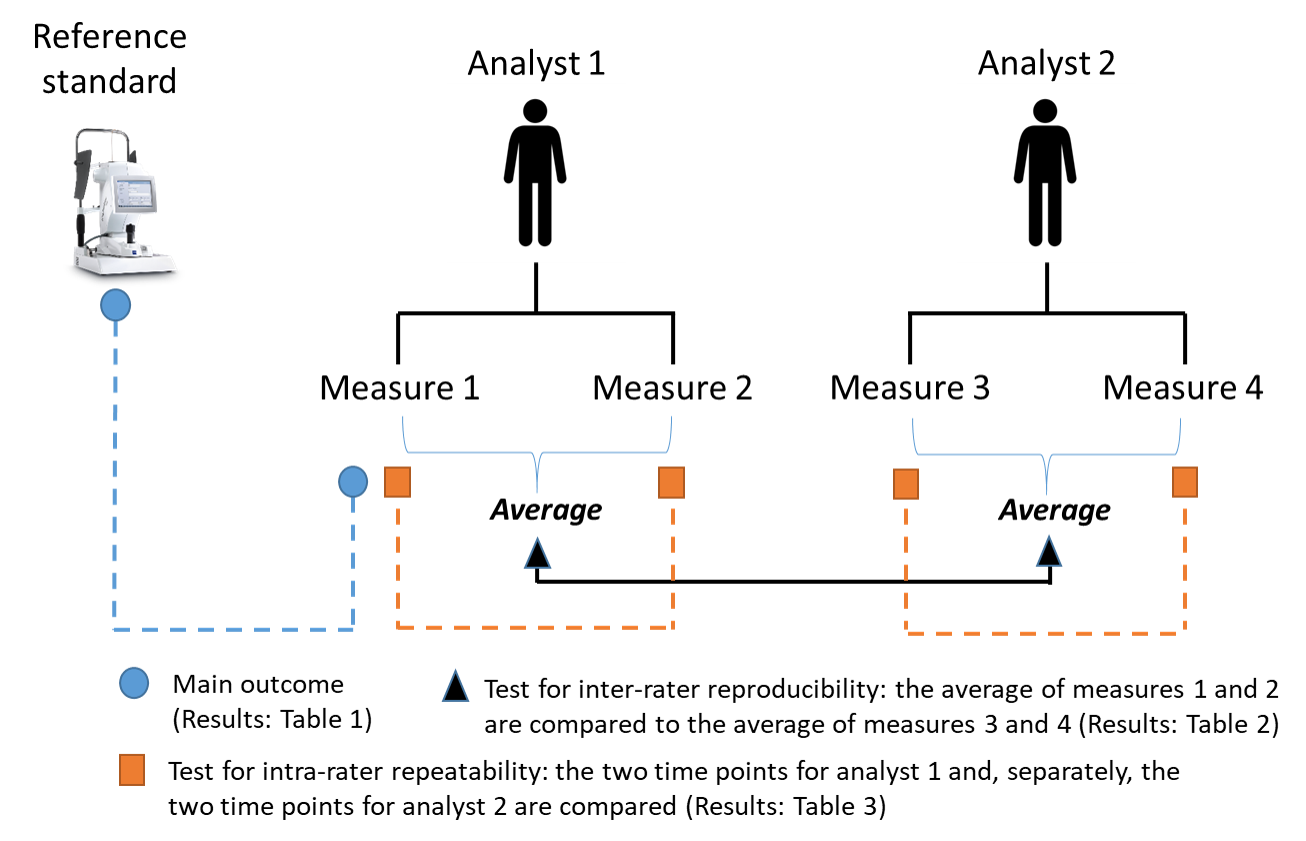

Supplement: Supplementary file 1 — Additional file 1. [file 12886_2022_2289_MOESM1_ESM.png]

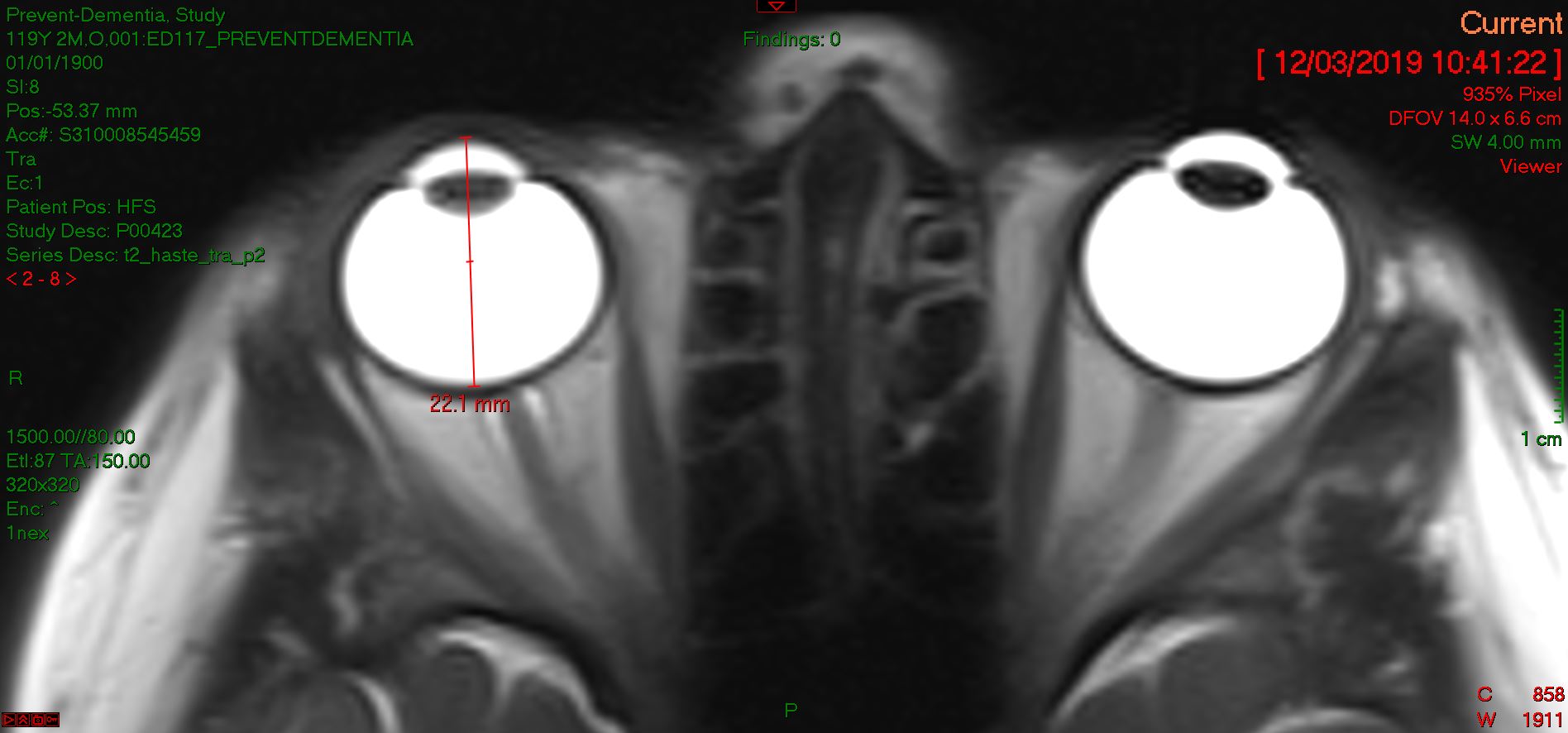

Supplement: Supplementary file 2 — Additional file 2. [file 12886_2022_2289_MOESM2_ESM.jpg]

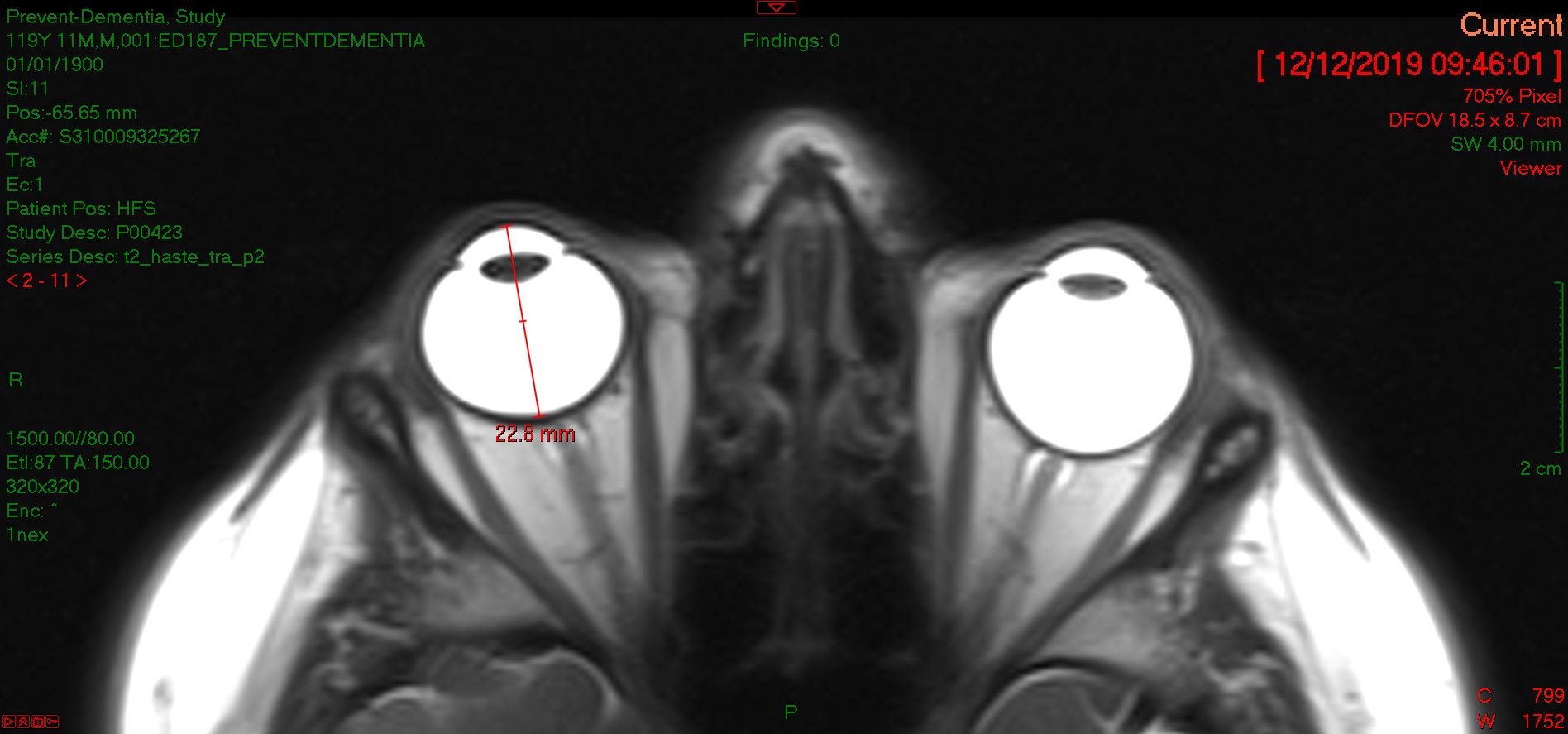

Supplement: Supplementary file 3 — Additional file 3. [file 12886_2022_2289_MOESM3_ESM.jpg]

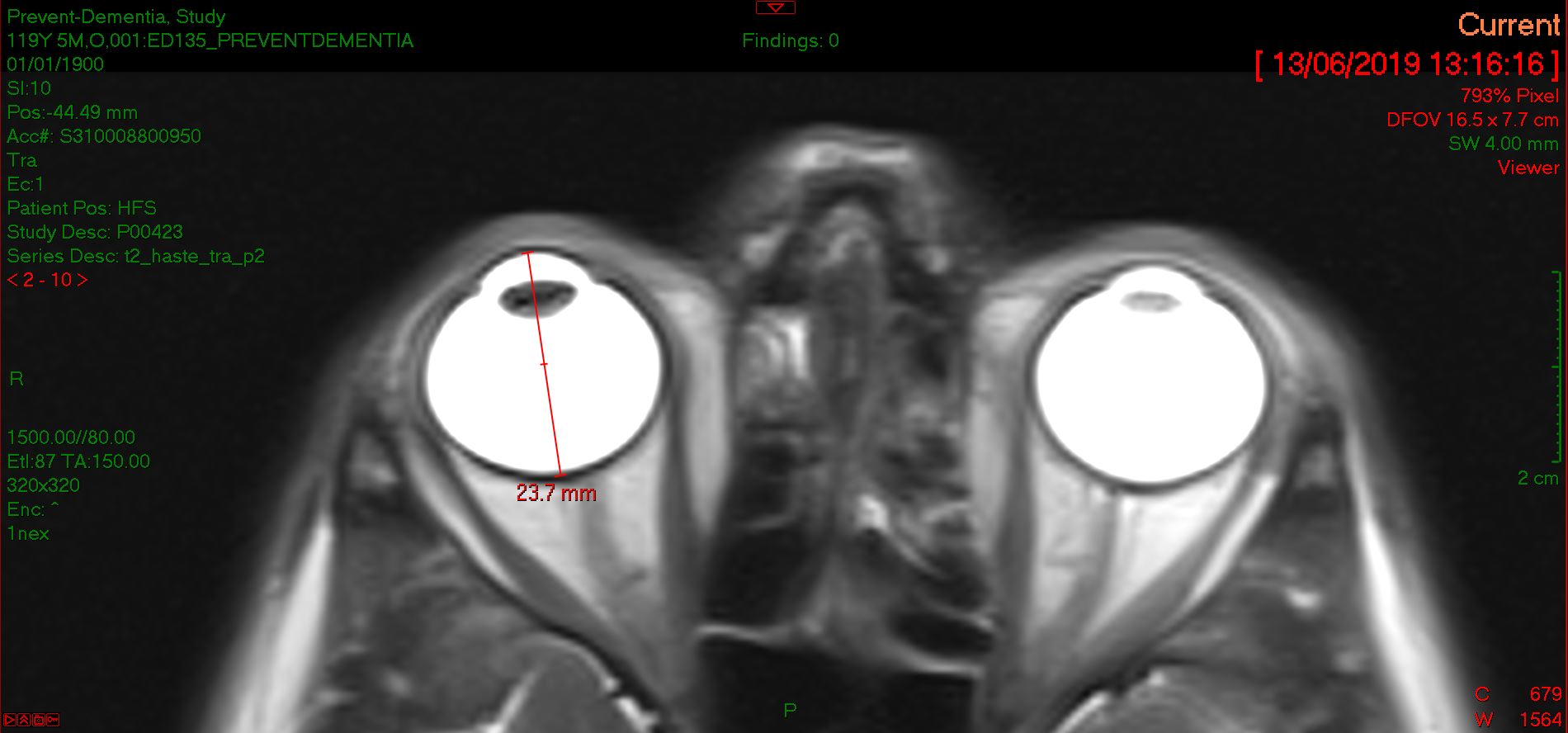

Supplement: Supplementary file 4 — Additional file 4. [file 12886_2022_2289_MOESM4_ESM.jpg]

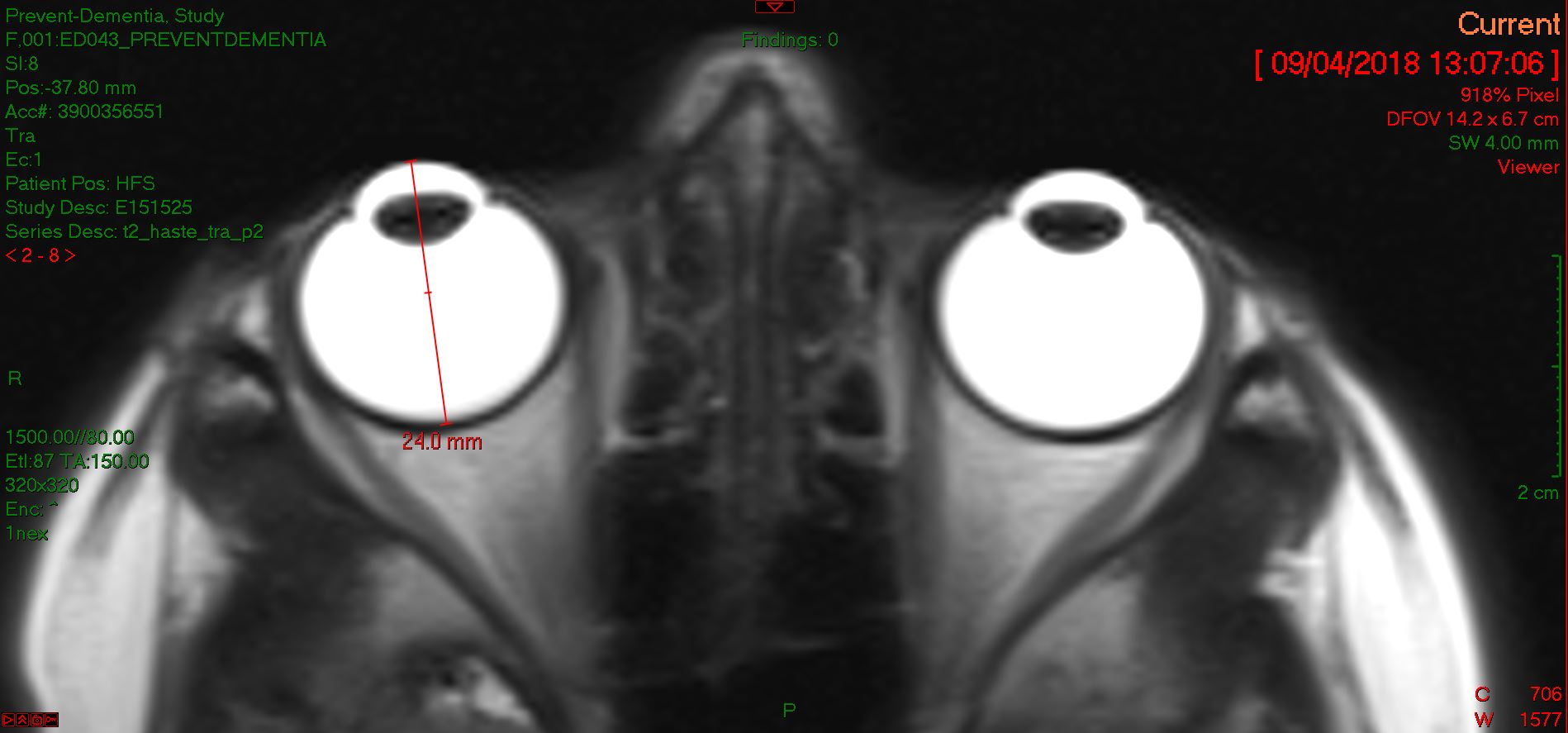

Supplement: Supplementary file 5 — Additional file 5. [file 12886_2022_2289_MOESM5_ESM.jpg]

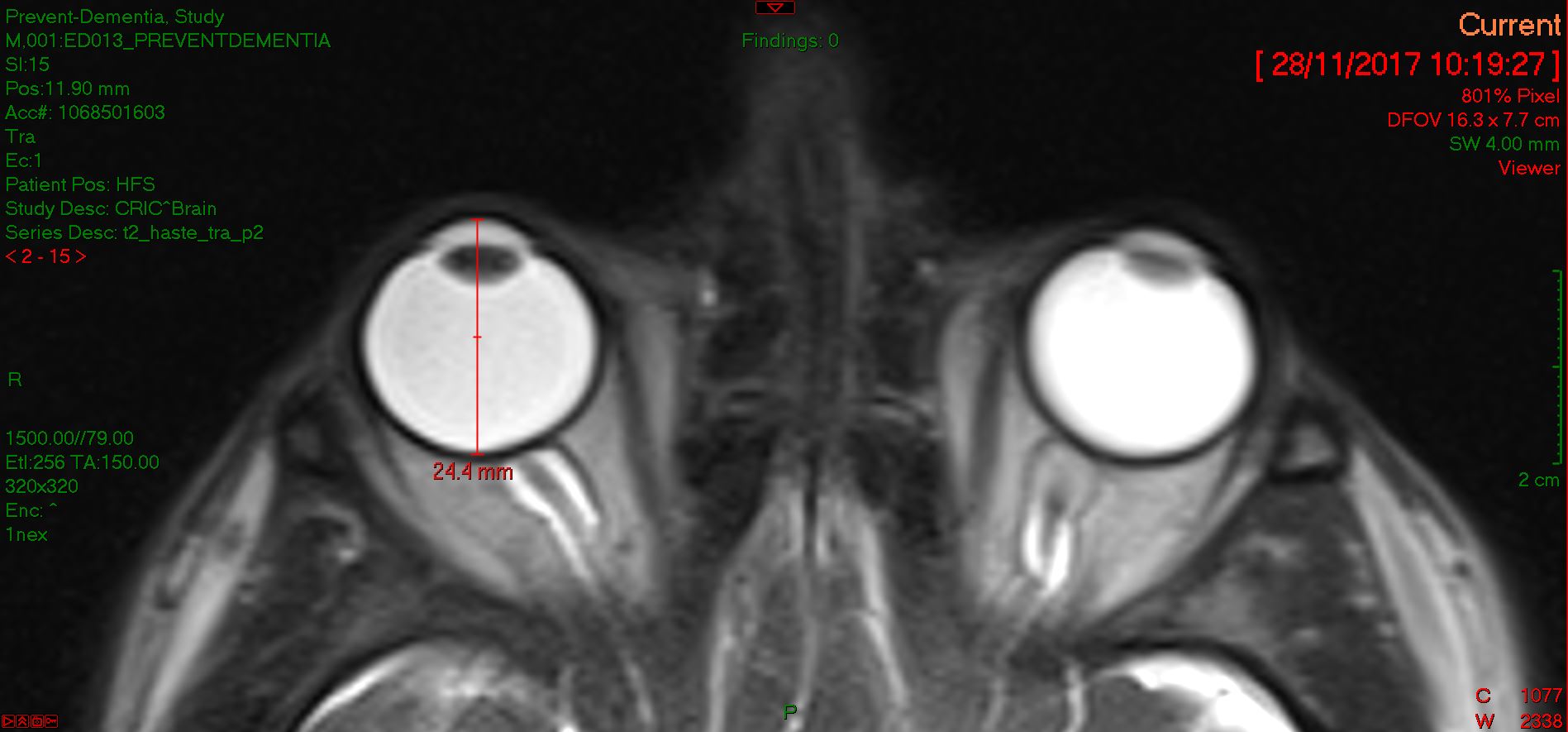

Supplement: Supplementary file 6 — Additional file 6. [file 12886_2022_2289_MOESM6_ESM.jpg]

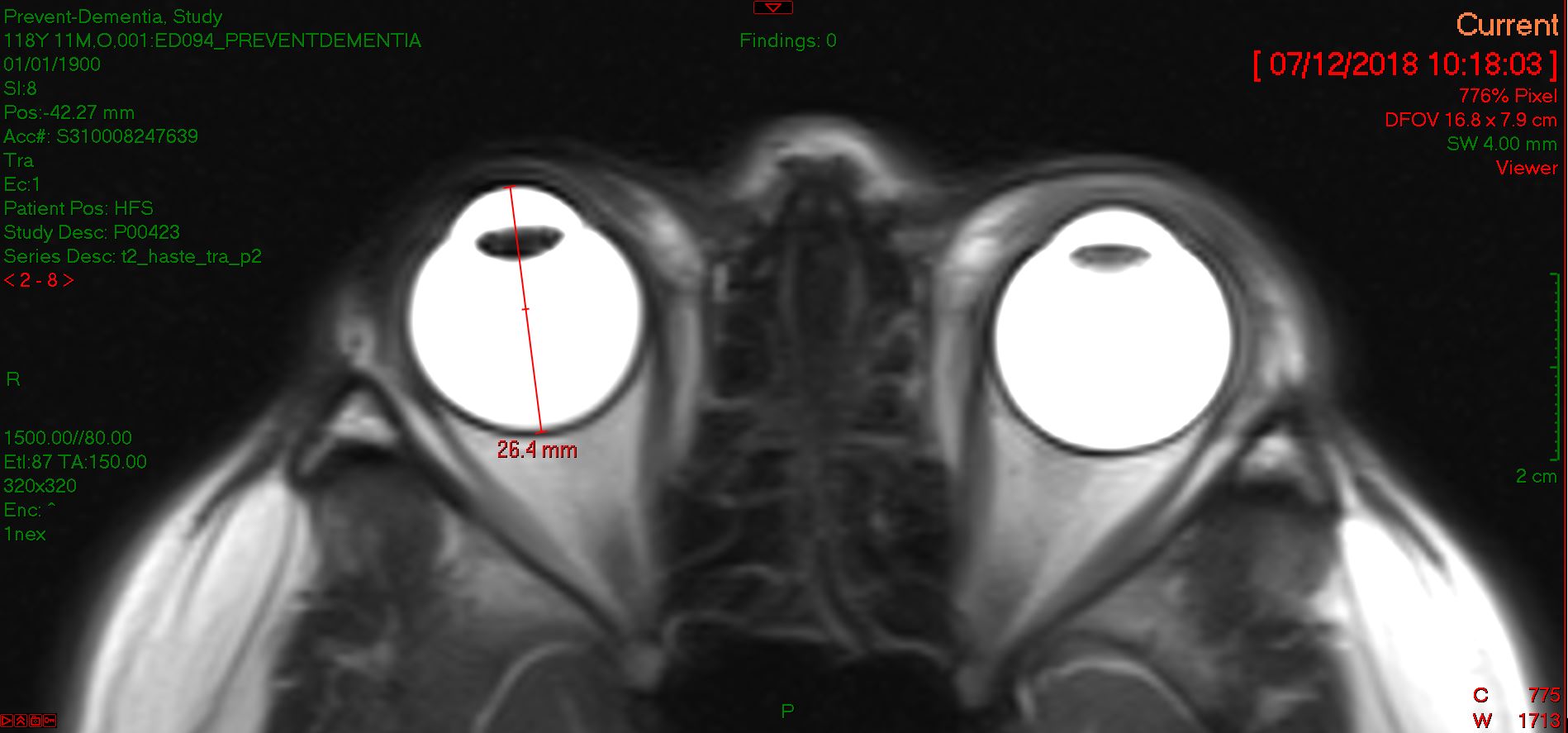

Supplement: Supplementary file 7 — Additional file 7. [file 12886_2022_2289_MOESM7_ESM.jpg]

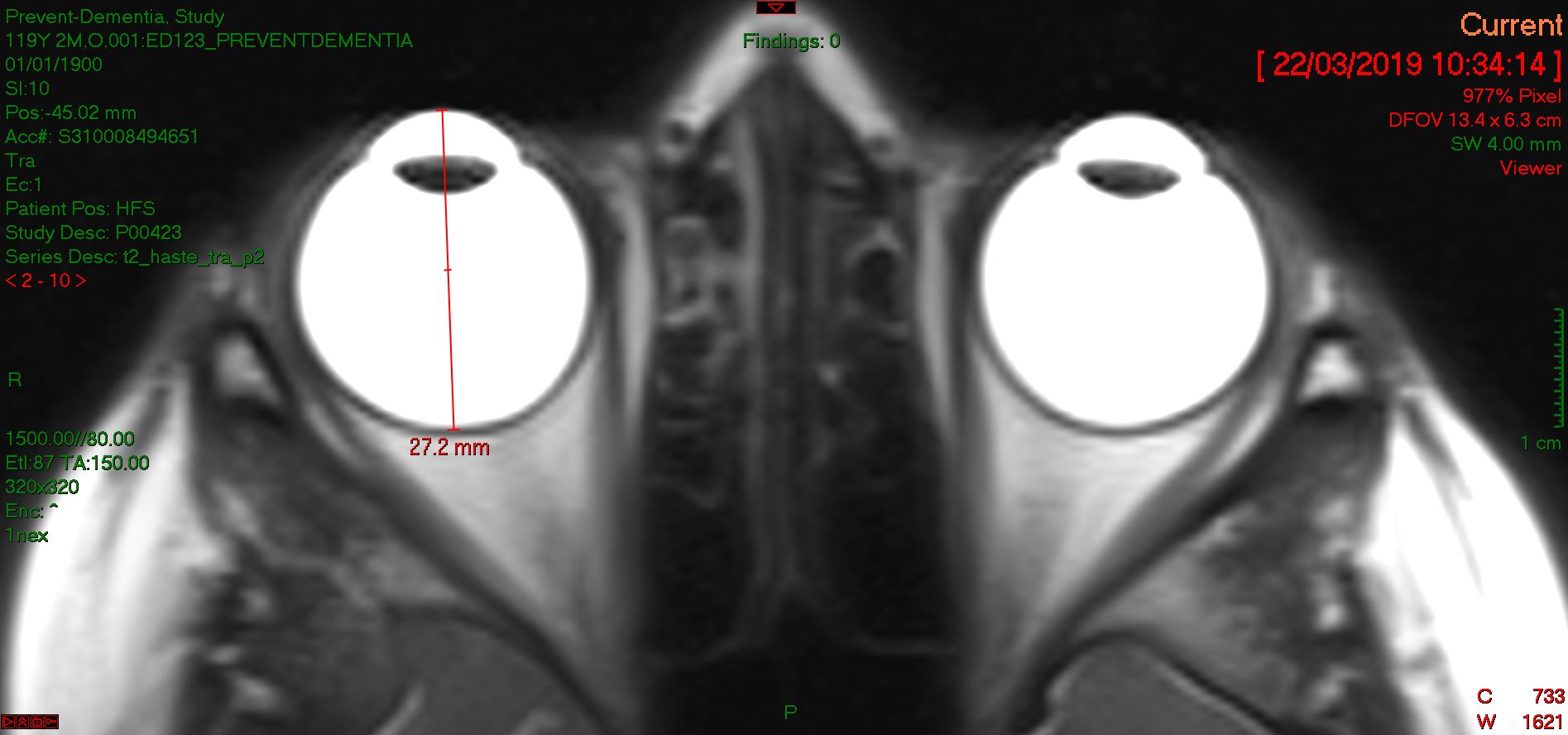

Supplement: Supplementary file 8 — Additional file 8. [file 12886_2022_2289_MOESM8_ESM.jpg]
